# A biomarker-validated time scale in years of disease progression has identified early- and late-onset subgroups in sporadic Alzheimer’s disease

**DOI:** 10.1186/s13195-023-01231-8

**Published:** 2023-05-02

**Authors:** Ove Almkvist, Agneta Nordberg

**Affiliations:** 1 Division of Clinical Geriatrics, Department of Neurobiology Care Sciences and Society, Karolinska Institutet, Stockholm, Sweden; 2 Theme Inflammation and Aging, Karolinska University Hospital, Stockholm, Sweden; 3 Department of Psychology, Stockholm University, Stockholm, Sweden

**Keywords:** Alzheimer’s disease, Time scale, Progression, Disease onset, Cognition, EOAD, LOAD

## Abstract

**Background:**

It is possible to calculate the number of years to the expected clinical onset (YECO) of autosomal-dominant Alzheimer’s disease (adAD). A similar time scale is lacking for sporadic Alzheimer’s disease (sAD). The purpose was to design and validate a time scale in YECO for patients with sAD in relation to CSF and PET biomarkers.

**Methods:**

Patients diagnosed with Alzheimer’s disease (AD, *n* = 48) or mild cognitive impairment (MCI, *n* = 46) participated in the study. They underwent a standardized clinical examination at the Memory clinic, Karolinska University Hospital, Stockholm, Sweden, which included present and previous medical history, laboratory screening, cognitive assessment, CSF biomarkers (Aβ_42_, total-tau, and p-tau), and an MRI of the brain. They were also assessed with two PET tracers, ^11^C-Pittsburgh compound B and ^18^F-fluorodeoxyglucose. Assuming concordance of cognitive decline in sAD and adAD, YECO for these patients was calculated using equations for the relationship between cognitive performance, YECO, and years of education in adAD (Almkvist et al. J Int Neuropsychol Soc 23:195-203, 2017).

**Results:**

The mean current point of disease progression was 3.2 years after the estimated clinical onset in patients with sAD and 3.4 years prior to the estimated clinical onset in patients with MCI, as indicated by the median YECO from five cognitive tests. The associations between YECO and biomarkers were significant, while those between chronological age and biomarkers were nonsignificant. The estimated disease onset (chronological age minus YECO) followed a bimodal distribution with frequency maxima before (early-onset) and after (late-onset) 65 years of age. The early- and late-onset subgroups differed significantly in biomarkers and cognition, but after control for YECO, this difference disappeared for all except the APOE e4 gene (more frequent in early- than in late-onset).

**Conclusions:**

A novel time scale in years of disease progression based on cognition was designed and validated in patients with AD using CSF and PET biomarkers. Two early- and late-disease onset subgroups were identified differing with respect to APOE e4.

**Supplementary Information:**

The online version contains supplementary material available at 10.1186/s13195-023-01231-8.

## Background

A small proportion of patients with Alzheimer’s disease (AD) carry autosomal-dominant mutations in the APP, PSEN1, or PSEN2 genes (adAD) [1]. For these individuals, the onset of disease typically occurs early in life, before 65 years of age [2, 3]. However, the genetic background of most patients diagnosed with sporadic AD (sAD) is unknown. The onset of disease varies from early to late in life. The mechanisms involved in disease development in these individuals could involve gene–gene or gene-environment interactions, comorbidity, lifestyle choices, resilience, or compensation [1, 4].

Although adAD and sAD patients generally vary in age of onset and details of clinical expression, they share similarities in the development of neuropathological features such as neuronal loss, amyloid plaques, and neurofibrillary and tau loads [5–8]. Individuals with adAD or sAD can also share clinical characteristics such as mode of onset, type of symptoms, progression, duration details [9, 10], patterns of brain atrophy [11, 12] and connectivity [13], as well as levels of biomarkers for the disease such as CSF biomarker levels of beta-amyloid (Aβ_42_), total-tau, and phosphorylated tau (p-tau) [14]. Subsequently, adAD and sAD are thought to be variants of the same biological disease. In fact, the clinical diagnoses of sAD and adAD follow the same criteria, as expressed in the National Institute of Neurological and Communicative Disorders and Stroke, and Alzheimer’s Disease and Related Disorders Association (NINCDS-ADRDA) manual [15], the Diagnostic and Statistical Manual of Mental Disorders (DSM-V) [16] and the International Classification of Diseases (ICD-10) [17]. In recent years, the NINCDS-ADRDA criteria have been challenged by biomarker-based criteria such as the NII-AA [18] and the IWG-2 [19] criteria.

In patients carrying the mutations for adAD, it is possible to calculate the years to the estimated clinical onset (YECO) of disease [20, 21] using the subject’s present age minus the family-specific age of onset of adAD obtained from medical files for individuals of the specific family. This method has made it possible to devise a general disease-onset time scale for adAD. This type of time scale has proven reliable, has been validated in relation to biomarkers of disease development, and has been successfully used in adAD research [20, 22–26].

In previous sAD research, time scales of disease progression have been designed from cross-sectional data or short-term changes in sAD patients. In a recent example, short-term changes in CSF biomarkers, PET ^18^F-fluorodeoxyglucose (FDG) metabolism, and cognition (global and episodic memory) were used to predict longitudinal trajectories in patients with sAD [27]. The results showed that early changes in episodic memory, hippocampal volume, and CSF biomarkers (Aβ_42_ and p-tau) were best fitted to a model of the time course of disease. A similar study showed that short-term changes from mild cognitive impairment (MCI) to AD were reliably predicted by changes in visuoconstructive performance, hippocampal volume, and FDG PET results [28]. In another approach, a cross-sectional study used machine learning with combined multimodal brain MR, CSF, and PET measures in patients with adAD to predict disease progression in a second sample of sAD patients [29]. Based on the large number of predictors and covariates (age, APOE status, current diagnostic state, and the time interval between clinical visits), the probability of reaching a more advanced state was modeled in cognitively normal, MCI, and AD individuals. Results established a complex pattern of preclinical changes and the clinical outcome [30]. To date, both quantitative measures (various biomarkers) and qualitative data (clinical stages, ATN nomenclature [31] including amyloid/tau/neurodegeneration) have been used to predict future status in AD. In addition, the traditional evaluation of symptom onset and duration of symptoms has recently been reviewed [32]; it was found that estimates of disease duration (before as well as after diagnosis) vary considerably, which hampers the drawing of reliable conclusions. In all the reported studies, the common denominator dealt with describing or predicting the expression of sAD during disease progression have used various system constructs (molecular, cell, tissue, brain, and human function), in a similar vein to the methods used to describe the temporal continuum of biological aging [33].

In this study, the main objective was to design an objective cognition-based time scale in years of disease progression for sAD using data on the decline in cognitive function but also taking cognitive reserve into account (in this case, years of education) [4]. The second aim was to validate the time scale in patients with sAD in relation to quantitative measures such as CSF beta-amyloid, p-tau, and t-tau [34]; PET ^11^C-Pittsburgh compound B (PiB) beta-amyloid and FDG metabolism [35]; and ATN framework [31].

## Methods

### Participants

The participants in this study were recruited from patients at the Memory Clinic, Karolinska University Hospital, Stockholm, Sweden, who had participated in PET research regarding beta-amyloid and glucose metabolism [36]. One group was diagnosed with MCI (*n* = 46) and another with AD (*n* = 48). Initially, all participants were examined according to a standardized comprehensive clinical procedure (see below) that did not include PET examination. The exclusion criteria were alcohol and drug abuse and psychiatric disease. Patients with marked cerebrovascular burden verified in the clinical examination were excluded as well.

The participants were subdivided into amyloid pathology using PET PiB cut-off (positive if neocortical PiB ≥ 1.41 and negative if neocortical PiB ≤ 1.40) resulting into four subgroups: PiB + AD (*n* = 40), PiB + MCI (*n* = 25), PiB − AD (*n* = 8), and PiB − MCI (*n* = 21). The PiB + subgroups can be understood as Alzheimer’s disease [31] and PiB − subgroups as non-AD pathologic change [31].

### Clinical examination

The clinical examination included medical history; a somatic, neurological, and psychiatric examination; cognitive screening with MMSE; an interview with a close informant; cognitive assessment (see below); routine analyses of blood, urine, and CSF (Aβ_42_, total-tau, and p-tau); MR imaging of the brain to evaluate the degree of atrophy (general, medial temporal, frontal and posterior) and other brain abnormalities.

### Diagnosis

The clinical diagnosis was decided at a consensus meeting of medical professionals (geriatricians, neurologists, psychologists, and nurses) and was based on all available examination reports except PET imaging. The dementia diagnosis followed the classical criteria of the DSM-V [37], and the NINCDS-ADRDA [15] as well as modified criteria that included CSF biomarkers [18, 19]. The MCI diagnosis was made according to the revised Petersen criteria [38].

### CSF biomarker levels

CSF levels of beta-amyloid, p-tau, and total-tau were included in the standard clinical protocol and measured as part of the clinical evaluation of the patients as described in detail in previous research [34]. The epitope of p-tau was 181. Abnormality was defined by the following cut-off values: beta-amyloid < 450 pg/mL, p-tau > 60 pg/mL, and total-tau > 400 pg/mL.

### Regional PET examination of PiB and FDG

The PET examinations were carried out at the Uppsala PET center within a few months of the clinical examinations; they covered 13 regions and measured PiB amyloid and FDG metabolism as described in previous publications [36]. The PET neocortical PiB value was used to classify the participants into amyloid-positive (≥ 1.41) and amyloid-negative (≤ 1.40) groups, as previously recorded [36]. The measurement of glucose metabolism used an index of aggregated values in the temporal, parietal, and posterior gyrus cinguli regions; abnormality was defined according to cut-off values for the index: positive (≤ 1.50) and negative (≥ 1.51) [39].

### Assessment of cognitive function

The standard clinical assessment of cognition included current global cognitive function, based on five subtests (the Information, Digit Span, Similarities, Block Design, and Digit Symbol tests) from the Wechsler Adult Intelligence Scale Revised [40, 41]. Short-term memory/attention was assessed using the Digit Span Forward test and the Corsi Span test [42]. The total score on the Rey Auditory Verbal Learning (RAVL)[42] test was used to assess verbal learning and 30 min retention in episodic memory. The Rey-Osterrieth 30 min retention test (RO retention) [42] was used to assess visuospatial episodic memory. Executive function was assessed using the Digit Symbol and Trail Making tests (TMTA and TMTB) [42]. Raw scores were converted to z-scores using a reference group of healthy adults at Karolinska University Hospital [43].

### Years to estimated clinical onset (YECO)

For each participant, the YECO were calculated using the equations obtained in a previous study of patients with adAD [20]. These equations were obtained for each cognitive test in carriers of five mutations associated with adAD [20]; they described the relationship between the test performance and three predictors: linear and quadratic YECO and years of education. The same three predictors and the associated beta weights were used in the present study, together with the cognitive test results, to find the unknown YECO in patients with AD or MCI. The median YECO was estimated from the five AD-sensitive tests (Similarities, Block Design, RAVL learning, RO retention, and Digit symbol) [20]. The concept of YECO has been shown to be valid and reliable in previous research in adAD [21, 44]

### Statistical analysis

Descriptive statistics and t-tests were used to analyze the baseline information. The formulas from the previous study of patients with adAD on the relationships between cognitive test results and linear and curvilinear YECO were used, along with years of education to represent cognitive reserve [20]: cognitive test result (raw score) = beta weight × YECO + beta weight × YECO^2^ + beta weight × years of education. The beta weights were taken from the previous study and the test results were from the present study, while YECO was unknown. YECO was obtained as the two roots of the equation, negative if the current stage of disease progression was prior to the estimated clinical onset (preclinical stage) and positive if the current stage of disease progression was later than the estimated clinical onset (clinical stage).

The validity of YECO as a marker of disease progression was evaluated by means of the association between YECO and the investigated biomarkers in PET and CSF, as assessed using Pearson correlation coefficients. These values were compared with the corresponding values for chronological age vs biomarkers in PET and CSF. A second validation was based on the ATN framework, using clinical cut-off values for binarization of all five biomarkers (PET PiB and FDG index, CSF Aβ_42_, total-tau, and p-tau) as normal or abnormal and binarization of YECO as negative or positive. The strength of association was expressed as the phi (ϕ) correlation coefficient together with *p*-values in *χ*^2^-statistics.

The estimated age of disease onset for each participant was calculated as their current age minus the median YECO obtained from five cognitive tests, in agreement with corresponding calculations in patients with adAD. A *χ*^2^-test was used to check whether the distribution of age at disease onset was normal. A *χ*^2^-test was used also used to analyze the association between early- vs late-onset and amyloid abnormality.

A k-means cluster analysis was applied to the median age at disease onset assuming two clusters, because the frequency distribution of age at disease onset was evaluated as bimodal showing two subgroups, one with early-onset and a second with late-onset disease.

The difference in biomarker levels between the early- and late-onset subgroups was analyzed using a *t*-test with and without control for the stage of disease progression (YECO) using covariance analyses.

## Results

### Characterization of the sample

In Table 1, the demographic characteristic (chronological age, sex, years of education) are presented for the two diagnostic groups (AD and MCI) subdivided according to beta-amyloid abnormality (positive if neocortical PiB ≥ 1.41 and negative if neocortical PiB ≤ 1.40). Two-way ANOVAs [diagnosis (MCI vs AD) and amyloid abnormality (yes vs no)] showed that the diagnostic and amyloid abnormality subgroups were comparable with respect to demographic characteristics (age, sex, years of education; all *p*’s > 0.1).

**Table 1:** Demographic (age, YECO, sex, and years of education) and basic clinical characteristics of patients with MCI or sAD subdivided according to amyloid abnormality (yes or no). *P*-values of two-way (diagnostic group and amyloid abnormality) ANOVAs

	MCI		AD		*p*		
	PiB +	PiB −	PiB +	PiB −	D	A	DxA
N (% females)	25 (56)	21 (62)	42 (57)	6 (33)	ns	ns	ns
Age, years	65.0 ± 7.5	63.1 ± 8.1	67.0 ± 8.7	65.4 ± 8.3	ns	ns	ns
Education, years	13.3 ± 3.5	12.4 ± 3.2	12.4 ± 3.8	12.7 ± 4.0	ns	ns	ns
YECO, years	− 1.2 ± 4.4	− 5.5 ± 5.6	+ 3.8 ± 4.6	+ 0.5 ± 5.8	***	**	ns
MMSE, score	27.5 ± 2.2	27.7 ± 2.7	24.6 ± 3.6	26.2 ± 2.7	**	ns	ns
APOE e4 + , *n* (%)	0.87 ± 0.82	0.70 ± 0.66	1.12 ± 0.78	1.33 ± 0.82	*	ns	ns
CSF Aβ_42_	569 ± 161	724 ± 285	418 ± 135	399 ± 122	***	ns	ns
CSF total-tau	455 ± 157	325 ± 185	615 ± 266	474 ± 333	*	*	ns
CSF p-tau	75.3 ± 23.0	56.5 ± 22.7	89.0 ± 31.2	63.0 ± 0.0	ns	ns	ns

*A*, amyloid abnormality; *Aβ*_*42*_, 42 amino-variant of beta-amyloid; *AD*, Alzheimer’s disease; *D*, diagnostic group; *MCI*, mild cognitive impairment; *MMSE*, Mini-Mental State Examination; *PiB*, ^11^C-Pittsburgh compound B; *p-tau*, phosphorylated tau

^*^ *p* < 0.05; ** *p*<0.01; *** *p* < 0.001

In contrast, the diagnostic and amyloid subgroups differed significantly in most clinical characteristics (YECO, MMSE, APOE, CSF Aβ_42_, total-tau; *p*’s > 0.001) due to diagnosis (*p*’s > 0.1), see Table 1. The main effect of diagnosis was due to significantly earlier disease onset in MCI than AD, higher score on MMSE in MCI than AD, lower presence of APOE e4 in MCI than AD, higher CSF Aβ_42_ in MCI than AD, and lower CSF total-tau in MCI than AD (all *p*’s < 0.001). There was one exception to this pattern of results, there was no significant effect on CSF p-tau due to diagnosis, amyloid, or diagnosis-by-amyloid interaction (*p*’s > 0.1). The main effect of amyloid abnormality was significant on YECO (*p* < 0.01) due to an earlier disease onset in PiB + patients compared to PiB − patients. The main effect of amyloid on CSF total-tau (*p* < 0.5) was caused by higher total-tau in PiB + patients compared to PiB − patients. There were no significant diagnosis-by-amyloid interaction effects (*p*’s > 0.1). These results indicate that the four subgroups were comparable in demographics, while clinical characteristics differed between MCI and AD patients. Amyloid positivity had a negative influence on disease progression (YECO); PiB + patients were closer to disease onset than PiB − patients. The neurodegeneration (CSF total-tau) was more pronounced in PiB + patients compared to PiB − patients.

### Design of the time scale for sAD

The previously obtained time scale for cognitive decline in relation to linear and curvilinear YECO and education in patients with adAD [20] was applied to the raw cognitive test score results for our patients along with their years of education, estimates of parameters for YECO and YECO^2^, and a constant for each of the five tests as follows:

$$\begin{array}{c}\mathrm{Similarities}=-0.552\times\mathrm{YECO}-0.013\times\mathrm{YECO}^{2}+0.511\times\mathrm{educ}.+\mathrm{constant}\;(9.624)\\\mathrm{Block}\;\mathrm{Design}=-1.441\times\mathrm{YECO}-0.027\times\mathrm{YECO}^{2}+1.274\times\mathrm{educ}.+\mathrm{constant}\;(5.971)\\\mathrm{RAV}\;\mathrm{Llearning}=-1.408\times\mathrm{YECO}-0.023\times\mathrm{YECO}^{2}+1.253\times\mathrm{educ}.+\mathrm{constant}\;(14.686)\\\mathrm{RO}\;\mathrm{retention}=-0.916\times\mathrm{YECO}-0.009\times\mathrm{YECO}^{2}-0.039\times\mathrm{educ}.+\mathrm{constant}\;(8.544)\\\mathrm{Digit}\;\mathrm{Symbol}=-1.573\times\mathrm{YECO}-0.024\times\mathrm{YECO}^{2}+2.594\times\mathrm{educ}.+\mathrm{constant}\;(3.473)\end{array}$$

The relationship between cognitive decline and the three predictors was significant in all five tests (p < 0.001: multiple *r*^2^ varied from 0.45 to 0.68; see Table 2). The linear negative YECO was significant for all five tests (*p* < 0.001), while the curvilinear YECO^2^ predictor was negative in all tests and significant in two tests (Similarities and Block Design; *p* < 0.05). Years of education was significant and positive in three tests (Block Design, RAVL learning, and Digit Symbol; *p* < 0.05).

**Table 2:** Results of multiple regression analyses with cognitive test performance over five tests as the dependent variable and years to estimated clinical onset (YECO and YECO^2^) and years of education as independent variables in adAD mutation carriers. Significant values are bolded. Note that results of performance are given as raw scores (the higher the better) for all tests

Test	*r*_mult_	*r*^2^	*F*	df	*p*	*β* (Y)	*β* (Y^2^)	*β* (educ)
Similarities	**0.67**	0.45	9.35	3/35	** < 0.001**	− 0.955^***^	− 0.540^*^	+ 0.211^ ns^
Block Design	**0.78**	0.61	19.13	3/36	** < 0.001**	− 1.034^***^	− 0.447^*^	+ 0.219^*^
RAVL learning	**0.81**	0.66	21.25	3/33	** < 0.001**	− 1.022^***^	− 0.399^ ns^	+ 0.218^*^
RO retention	**0.75**	0.56	13.38	3/32	** < 0.001**	− 0.945^***^	− 0.229^ ns^	− 0.010^ ns^
Digit Symbol	**0.82**	0.68	24.27	3/35	** < 0.001**	− 0.894^***^	− 0.328^ ns^	+ 0.311^**^

*ns*, not significant; *RAVL*, Rey auditory verbal learning; *RO*, Rey-Osterrieth

^*^ *p* < 0.05; ** *p*<0.01; *** *p* < 0.001

Next, the unknown YECO representing each individual patient’s position on the time scale was obtained from quadratic equations as two roots, real or imaginary, in each test. Imaginary roots and/or missing test results occurred most frequently in the Similarities test (29%) and least frequently in the Block design and RO retention tests (0%).

The median root value across the five tests showed that most MCI patients had not reached the estimated age of clinical onset when they were examined (YECO =  − 3.1), while most AD patients had passed the estimated age of clinical onset (YECO =  + 3.4). Although the median time point was close to the estimated onset, the scale varied from the early preclinical stage about 10 years prior to disease onset to moderately severe disease about 10 years after the estimated onset. The difference in YECO between diagnostic groups (MCI vs AD) was significant in four of five tests (Similarities, Block Design, RAVL learning, and RO retention; *p* < 0.5, *p* < 0.001, *p* < 0.01, and *p* < 0.001, respectively). The differences in YECO between amyloid abnormality subgroups (present vs absent) was significant in three tests (Similarities, RAVL learning, and Digit Symbol; *p* < 0.01, *p*’s < 0.05, respectively). The diagnostic group-by-amyloid subgroup interaction was not significant in any tests (*p* > 0.1). The estimates for YECO varied across the five cognitive tests. The performance in the Similarities test (verbal domain) indicated a time point years ahead of the estimated clinical onset of disease (YECO =  − 3.0) that was earlier in patients with MCI (YECO =  − 6.5) than in those with AD (YECO =  − 0.7), indicating relatively low sensitivity for this test. On the other hand, the performance in the Digit Symbol test (executive domain) indicated a time point in the clinical stage close to the estimated clinical onset of disease (YECO =  + 1.5) that was earlier in MCI patients (YECO =  − 1.4) than in AD patients (YECO =  + 4.0), indicating relatively good sensitivity. In Table 3, the median time of disease progression is presented for each of the five tests in MCI and AD patients divided into subgroups of PiB amyloid abnormality (positive or negative).

**Table 3:** The median years to estimated clinical onset (YECO) for each test in MCI and AD patients subdivided according to beta-amyloid abnormality (yes vs no). *P*-values for two-way (diagnostic group and amyloid abnormality) ANOVAs

Cognitive test, YECO	YECO				*p*		
	MCI		AD				
	PiB +	PiB −	PiB +	PiB −	D	A	DxA
Similarities	− 4.1	− 11.5	± 0.0	− 4.8	*	**	ns
Block Design	− 1.7	− 3.4	+ 4.9	+ 1.8	***	ns	ns
RAVL learning	− 1.4	− 6.6	+ 2.7	+ 0.7	**	*	ns
RO retention	− 4.0	− 8.6	+ 4.1	− 0.3	***	ns	ns
Digit Symbol	+ 1.4	− 5.1	+ 4.8	− 1.1	ns	*	ns
Mean YECO	− 1.2	− 5.51	+ 3.8	+ 0.5	***	**	ns

*A*, amyloid abnormality (yes vs no); *D*, diagnostic group; *DxA*, diagnostic-by-amyloid abnormality subgroup interaction; *ns*, nonsignificant; *RAVL*, Rey auditory verbal learning; *RO*, Rey-Osterrieth

^*^ *p* < 0.05; ** *p* < 0.01; *** *p* < 0.001

A two-way ANOVA using diagnostic group (MCI vs AD) and PiB amyloid abnormality (≥ 1.41 vs ≤ 1.40) as factors showed that the current time of disease advancement (YECO) differed significantly for diagnosis in four of five tests (Similarities, Block Design, RAVL learning and RO retention; *p* < 0.05, *p* < 0.001, *p* < 0.01, and *p* < 0.001, respectively); see Table 3). The effect of amyloid was significant in three of five tests (Similarities, RAVL learning, and Digit Symbol; *p* < 0.01, *p*’s < 0.05, respectively) and the overall mean (*p* < 0.01) due to PiB amyloid abnormality (*p* > 0.1). The diagnostic group-by-PiB amyloid interaction was also not significant in any test (*p* > 0.1).

### Validation of the time scale in relation to CSF and PET biomarkers

#### Quantitative data

Firstly, the validity of the time scale was evaluated using quantitative data on the time scale of disease course (YECO) and the CSF biomarkers (Aβ_42_, total-tau, and p-tau) and PET PiB and FDG index results expressed as correlations. These correlations were compared with the corresponding correlations for chronological age in all participants, and separately in the PET PiB amyloid subgroups (see Table 4). To summarize, the results for the age-related correlations with biomarkers were not significant in all patients and the subgroups of amyloid abnormality (*p*’s > 0.1).

**Table 4:** The correlation between chronological age and median years to estimated clinical onset (YECO) of patients in relation to CSF biomarkers (Aβ_42_, total-tau, and p-tau), PET PiB neocortical amyloid and PET FDG index in all patients for subgroups with and without beta-amyloid abnormality in PiB. Significant associations are bolded

Biomarker	All patients		Subgroups of amyloid abnormality			
	*n* = 94		yes (*n* = 67)		no (*n* = 27)	
	Age	YECO	Age	YECO	Age	YECO
CSF Aβ_42_	− 0.03	− 0.43^***^	− 0.01	− 0.42^***^	+ 0.02	− 0.24
CSF total-tau	− 0.00	+ 0.26^*^	− 0.04	+ 0.09	− 0.03	** + **0.20
CSF p-tau	+ 0.14	+ 0.06	+ 0.08	− 0.09	± 0.00	− 0.32
PET PiB neoctx	+ 0.14	+ 0.47^***^	+ 0.15	+ 0.19	− 0.09	** + **0.16
PET FDG index	− 0.05	− 0.63^***^	+ 0.29	− 0.40^*^	− 0.45	− 0.59^*^

*Aβ*_*42*_, 42 amino-variant of beta-amyloid; *FDG*, ^18^F-fluorodeoxyglucose; *neoctx*, neocortex; *PiB*, ^11^C-Pittsburgh compound B; *p-tau*, phosphorylated tau; *YECO*, years to estimated clinical onset

^*^ *p* < 0.05, ** *p* < 0.01, *** *p* < 0.001

In contrast, the median estimate of the current time of disease progression (YECO) was significantly associated with four of five biomarkers: CSF Aβ_42_ (decreasing Aβ_42_ linked to progression), CSF total-tau (increasing total-tau linked to progression), PET PiB abnormality (increasing PiB linked to progression) and PET FDG index (decreasing FDG index linked to progression) in all patients. The exception was a nonsignificant correlation for p-tau. Similar significant correlations were obtained for CSF Aβ_42_ (decreasing Aβ_42_ linked to progression) and PET FDG index (decreasing FDG index) in amyloid subgroups (see Table 4). Again, there were no significant correlations for p-tau. In summary, the YECO measure was favorable compared to chronological age. It is noteworthy that the correlation between YECO and chronological age was not significant (*r* = 0.15, *p* > 0.1, *n* = 94).

**Fig. 1:**
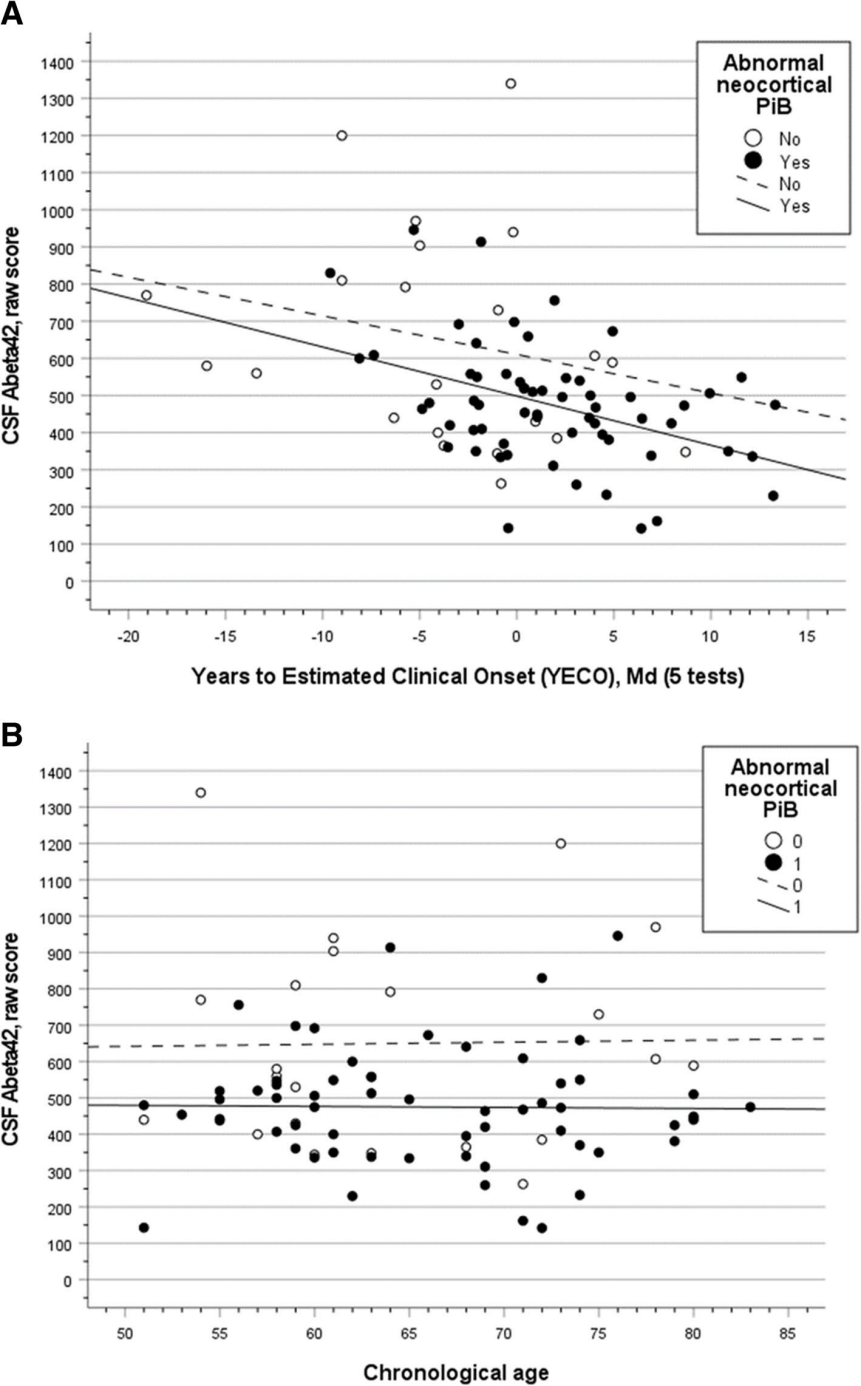
The relationship between CSF Aβ_42_ and years to estimated clinical onset (YECO) in patients with sAD or MCI with or without abnormal PET.^11^C-Pittsburgh compound B (PiB) amyloid levels (**A**); and between CSF Aβ_42_ levels and chronological age in sAD and MCI patients with or without abnormal PET PiB amyloid levels (**B**)

Figure 1 panel A shows the association between CSF Aβ_42_ and YECO in PET amyloid (positive and negative) subgroups patients. Panel B shows the corresponding association for CSF Aβ_42_ and chronological age. Basically, the figures demonstrate that increasing YECO was related to significantly increasing AD pathology shown as decreasing CSF Aβ_42_ in relation to increasing YECO in the PiB + subgroup (*r* = 0.42, *p* < 0.001, *n* = 61) and the PiB − subgroup (*r* =  − 0.24, *p* > 1, *n* = 22). The association between chronological age and CSF Aβ_42_ was not significant in PiB + subgroup (*r* =  − 0.01, *p* > 0.1, *n* = 67) or the PiB − subgroup (*r* =  + 0.02, *p* > 0.1, *n* = 22).

#### Qualitative data

A second evaluation of validity was performed using the ATN framework (A for amyloid, T for tau pathology, and N for neurodegeneration). Binary cut-off values as data (abnormal vs normal) were used for CSF biomarkers (Aβ_42_, total-tau, and p-tau) as well as PET PiB and PET FDG index, and corresponding binary data cut-off data for the time scale (YECO, negative vs positive). The phi correlation coefficients were significant for PET PiB (A in ATN; ϕ = 0.39, *p* < 0.001), CSF total-tau (N in ATN; ϕ = 0.24, *p* < 0.05), and FDG index (N in ATN; ϕ = 0.64, *p* < 0.001), but not in the other two biomarkers (CSF Aβ_42;_ A in ATN and CSF p-tau CSF, T in ATN; *p*’s > 0.1).

### Predicting the onset of disease in sAD patients

**Fig. 2:**
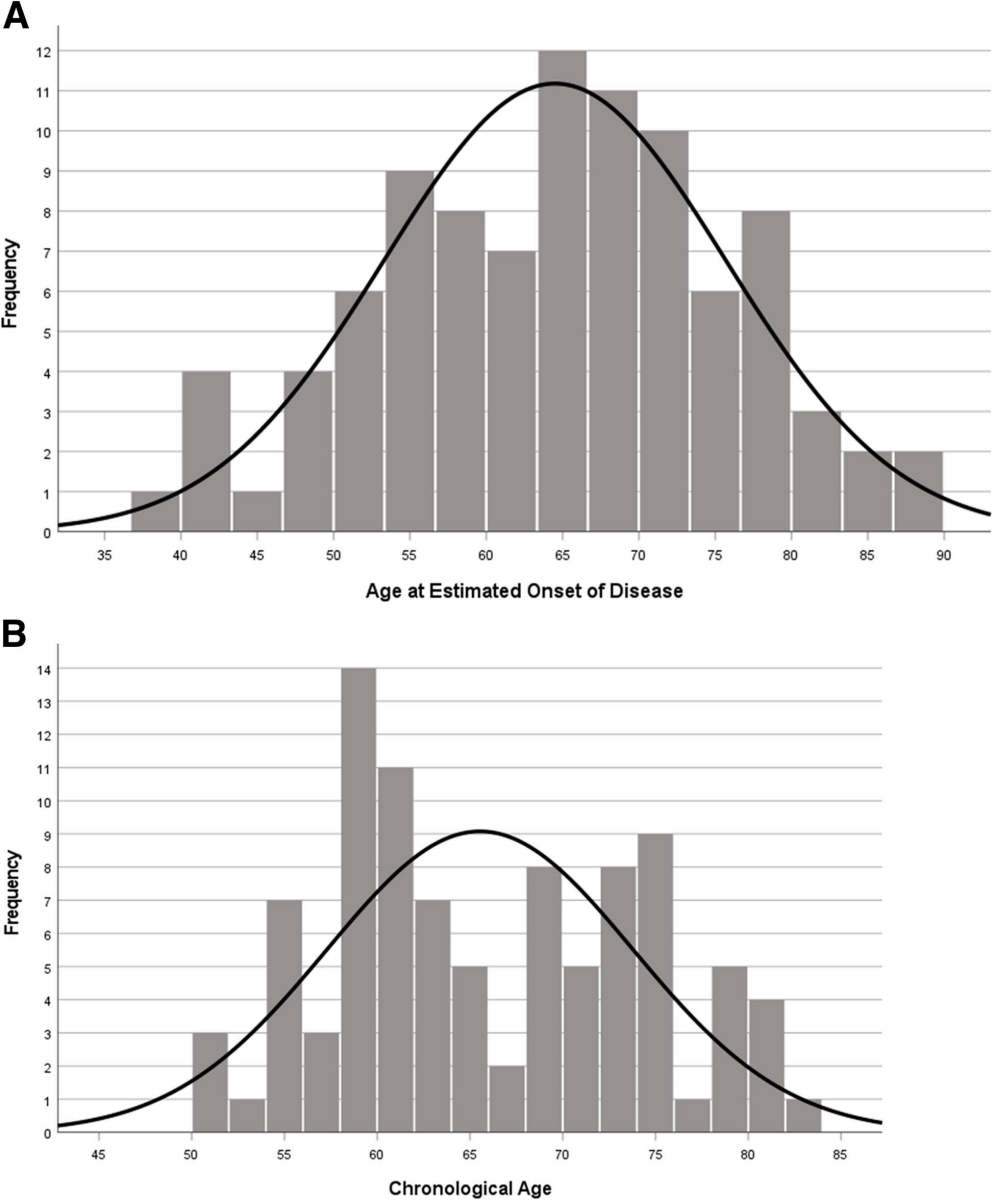
Histogram showing the distribution of median estimated age at onset of clinical sporadic Alzheimer’s disease (sAD) in all patients with MCI or sAD (**A**) and the corresponding distribution of chronological age (**B**)

The estimated age at onset of disease was obtained for each test for all individuals by calculating the difference between the individual’s chronological age and the number of years to the estimated clinical onset. Figure 2 panel A shows the distribution of the YECO-based median age at disease onset for all patients across the five tests. The hypothesis that the distribution deviated from a normal distribution was not rejected (*p* = 0.08), while the corresponding hypothesis for the distribution using chronological age was rejected (*p* < 0.001; see Fig. 2B). The chronological age data indicated the presence of two subgroups for disease onset, and this may also be the case for the YECO-based median age at disease onset data. A k-means cluster analysis of the chronological age at disease onset data showed that the cut-off between the subgroups of young (*n* = 52, M ± SD: 59.0 ± 3.8 years) and elderly (*n* = 42; M ± SD: 73.6 ± 4.1 years) individuals was at about 66 years. A similar cluster analysis of the YECO-based median age at disease onset data indicated a cut-off at 65 years of age between early (*n* = 54; M ± SD: 58.8 ± 4.7 years) and late-onset (*n* = 40; M ± SD: 70.5 ± 6.6 years) subgroups.

**Fig. 3:**
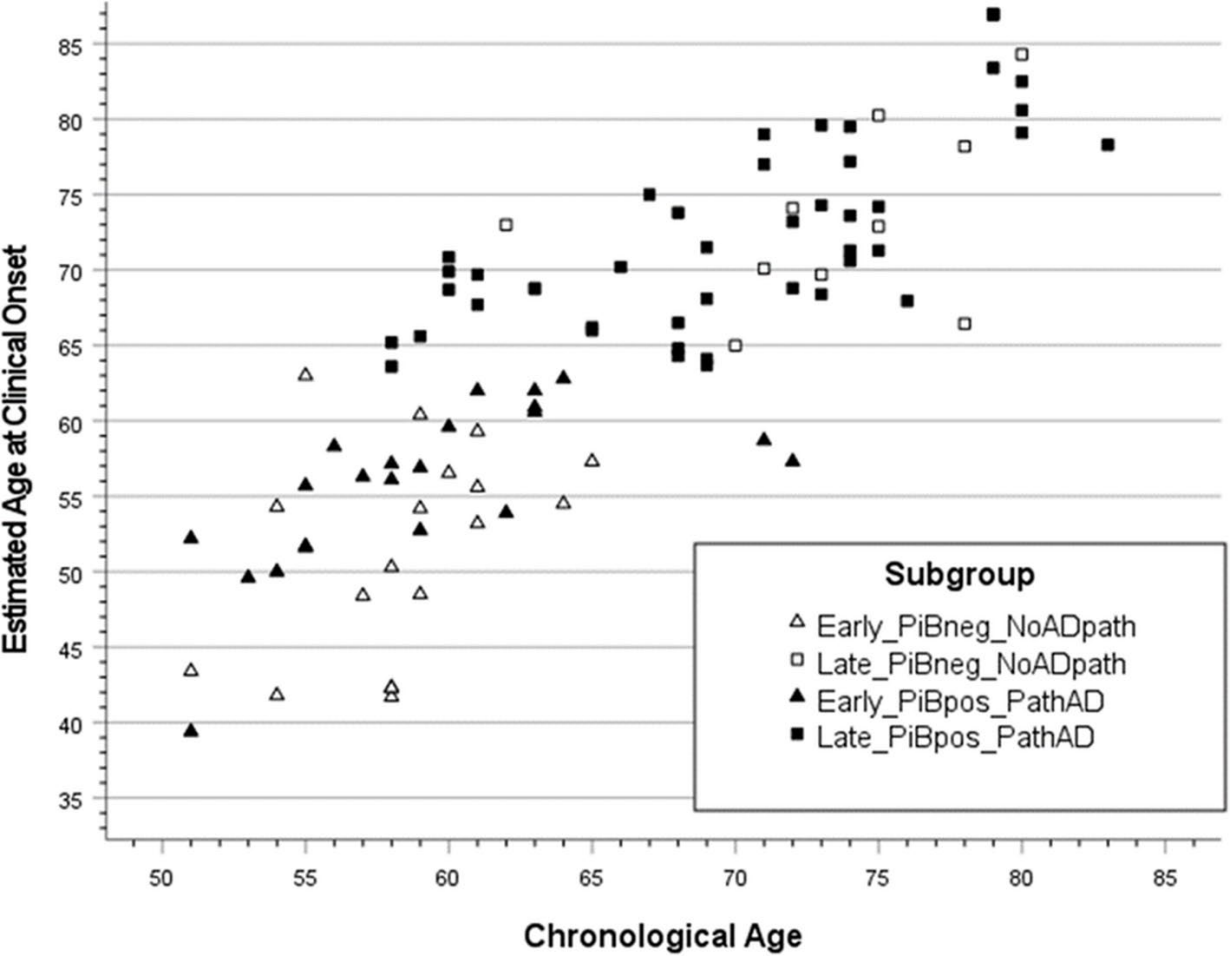
Scatter plot of the relationship between chronological age at onset of disease and age at estimated onset of disease in patients with early or late onset of disease

The relationship between the YECO-based median age at disease onset and chronological age at disease onset for subgroups with early and late disease onset is presented in Fig. 3. The corresponding figure for patients with a clinical diagnosis of MCI and AD is presented in the Supplement, Fig. 1. It is obvious that the data for early and late-onset as well as MCI and AD patients were scattered over chronological age and estimated age at onset of disease and the regression was close to linear. The early and late-onset patients were significantly different in composition of AD pathology (*χ*^2^ = 8.78, *p* < 0.01), the early-onset PiB + group (*n* = 27), the early-onset PiB − group (*n* = 20), the late-onset PiB + group (*n* = 40) and the late-onset PiB − group (*n* = 7) showing that late onset is associated with PiB abnormality. However, it has to be kept in mind that these data reflect an on-the-spot account.

The relevance of the early and late subgroups of disease onset was further analyzed without taking the current point of progression (YECO) into account, see Table 5. Results showed that early and late subgroups of disease onset differed significantly in CSF Aβ_42_, PET PiB, and FDG, and the five cognitive tests, showing advanced disease in the late-onset group (*p* < 0.05 or *p* < 0.01). Interestingly, these differences disappeared when YECO was introduced to control for the current time of progression, see Table 5. The YECO covariate was significant in all measures (*p* < 0.05 or stronger) except for CSF p-tau. Unexpectedly, the early and late subgroups differed according to the frequency of the APOE e4 allele (*p* < 0.01) even after the YECO control was introduced (*p* < 0.01). The frequency of the APOE e4 allele was higher in the early disease onset subgroup than in the late-onset subgroup. Corresponding analyses based on differentiating patients into young and elderly subgroups by CSF and PET biomarkers and cognition did not support any relevant biomarker association when patients were grouped by chronological age. Again, YECO was more favorable than chronological age as an index of progression.

**Table 5:** Descriptive data on APOE, CSF, and PET biomarkers, cognitive tests, and statistical outcome (*p*-values) in one-way (early vs late onset) ANOVA in patients with mild cognitive impairment or sporadic Alzheimer’s disease. Results are given with and without control for the current stage of progression (YECO) on APOE, CSF (Aβ_42_, total-tau, phosphorylated tau) and PET (neocortical PiB and FDG index) biomarkers, and five cognitive tests (Similarities, Block Design, RAVL learning, RO retention, and Digit Symbol)

Biomarker/test	Early	Late	No control	Control	
			E vs L	Covariate	E vs L
			*p*	*p*	*p*
APOE e4, proportion	1.1 ± 0.8	0.9 ± 0.8	ns	*	**
CSF Aβ_42_, pgm/mL	584 ± 235	458 ± 177	**	**	ns
CSF total-tau, pgm/mL	476 ± 265	521 ± 241	ns	*	ns
CSF p-tau, pgm/mL	74 ± 27	79 ± 31	ns	ns	ns
PET neocortical PiB	1.42 ± 0.23	1.58 ± 0.24	***	***	ns
PET FDG index	1.52 ± 0.39	1.32 ± 0.29	*	***	ns
Similarities, *z*-score	− 0.5 ± 1.2	− 1.2 ± 1.6	**	***	ns
Block Design, *z*-score	− 0.9 ± 0.9	− 1.7 ± 0.8	***	***	ns
RAVL, *z*-score	− 1.2 ± 1.4	− 2.3 ± 1.3	***	***	ns
RO retention, *z*-score	− 0.7 ± 1.1	− 1.3 ± 0.8	**	***	ns
Digit Symbol, *z*-score	− 1.1 ± 1.1	− 2.2 ± 1.2	***	***	ns

*Aβ*_*42*_, 42 amino variant of beta-amyloid; *E*, early onset; *FDG*, fluorodeoxyglucose; *L*, late onset; *PiB*, Pittsburgh compound B; *p-tau*, phosphorylated tau; *RAVL*, Rey auditory verbal learning; *RO*, Rey-Osterrieth

^*^ *p* < 0.05, ** *p* < 0.01, *** *p* < 0.001

## Discussion

### Design of the time scale

The study investigated the design of a time scale for disease progression in memory clinic patients with MCI and AD based on a previous study of patients with adAD [20]. This previous study presented data on the relationship between cognition and years to estimated clinical onset and years of education, which was used in the present study to calculate years to estimated clinical onset in the present cohort. It was assumed that the time-related decline in cognition will be similar in adAD and sAD patients [6, 8, 10, 13, 14, 26, 27]. The results of the current study support that YECO equations used in adAD families also worked well in patients with sAD.

The core feature of the time scale is that patients were assigned to a defined time of disease progression based on general cognitive performance (median of five AD-sensitive tests). The median value was preferred because (i) the mean could be influenced by extreme values in specific tests and (ii) the median provides a summary of cognition and not a domain-specific measure. The current time of disease progression was earlier in patients with MCI (mean − 3.1 years prior to the estimated clinical onset) than in those with AD (mean 3.4 years after the estimated disease onset) implying a difference in mean progression between MCI and AD patients amounting to about 6 years when measured by YECO (− 3.1 vs + 3.4; MCI and AD, respectively) to compare with a difference in chronological by less than 3 years (64.1 vs 66.9; MCI and AD, respectively). The estimated current time of progression was significantly affected by subgrouping the MCI patients into amyloid-positive with more advanced disease compared to amyloid-negative patients. This finding relates to the distinction between AD pathology and non-AD pathology [31]. The opposite trend was seen in AD patients, although it was not significant. This finding is problematic for the “biological definition” of AD [31]. The pattern of results indicates that disease progression rather than chronological age is the driving factor for disease development. Interestingly, there was no significant association between YECO and chronological age in the present cohort of patients. This finding may be pondered upon in light of recent research results from a very large cohort of individuals varying in cognitive status from normal aging to dementia as well as in age from 20 to 100 years [45].

### Validation of the time scale

The estimated clinical onset of adAD has been validated against the observed onset of disease in adAD patients and in the parents of adAD patients, with marked concordance [21, 44].

In this study, the time scale was quantitatively validated in relation to CSF and PET biomarker levels, i.e., a type of construct validity. The YECO time scale was significantly associated with CSF total-tau levels, PET PiB beta-amyloid levels, and FDG metabolism, in contrast to chronological age which had no significant association with the biomarkers. This pattern of results demonstrated that the YECO time scale is a biologically valid measure; in addition, it remained more valid than chronological age when all patients (MCI and sAD) were included. The same pattern was observed when patients were subdivided according to PET amyloid status (positive or negative). Interestingly, the association between CSF p-tau levels [34] and PET PiB abnormality was not significant, while the association between CSF p-tau levels and normal PET PiB was significant. The reason for this unexpected finding is not known. To speculate, the sample of patients may have been biased regarding CSF p-tau, as there were some missing data for the sample (*n* = 25, 27%) [36].

An alternative validation was performed by using the ATN framework. The outcome partly replicated the quantitative validation in that the time scale (YECO) was significantly associated with PET PiB amyloid (A in the ATN framework), CSF total-tau, and PET FDG metabolism (N in the ATN framework), and that there was no significant association with CSF p-tau (T in the ATN framework). This finding was unexpected, see the previous paragraph.

In previous research, the progression of the disease has been studied by measuring biomarkers of MR, CSF, and PET in adAD patients and then applying the results to outcome measures in sAD patients [27–30].

### Onset of disease in sAD

A spin-off effect of the time scale was the possibility of predicting the onset of disease based on the difference between the patient’s chronological age and YECO. The distribution was interpreted as bimodal, with one maximum before 65 years of age and a second maximum after 65 years of age. This interpretation indicated two subgroups with different onsets of disease. One subgroup (*n* = 47, 22 were PiB + and 18 were PiB −) with an early onset was most frequently diagnosed with MCI. T The mean age at onset was 60 ± 5 years. The majority of the second subgroup with a relatively late onset was most frequently diagnosed with AD (*n* = 47, 43 were PiB + and 11 were PiB −) and the mean age at onset was 71 ± 6 years). There were no differences in the demographics of the subgroups except for age, but they differed significantly in some biomarkers (CSF Aβ_42_, PET PiB, and FDG index) and in cognitive performance, indicating more severe disease in the late-onset group. However, the significant differences disappeared when the patient’s current position on the timeline was used as a covariate, implying that YECO, and not chronological age, is the driving factor for disease status. It is a challenge to understand the reasons for the two subtypes differing in disease onset. The clue may lie in the APOE e4 allele being significantly more frequent in the early-onset group than in the late-onset group. Similar results have been reported previously [46, 47]. Other researchers have found that plasma proteins associated with degeneration (GFAP and NfL) are elevated in early-onset AD compared to late-onset AD [48]. In addition, it is well known that APOE e4 heterogeneity or homogeneity bring forward the time of a dementia diagnosis in AD patients [49].

The validity of the two subgroups in age of disease onset is supported by evidence in previous research regarding early-onset AD (EOAD) and late-onset AD (LOAD) [50]. In that review, it was concluded that EOAD (onset before age 65) and LOAD (onset after age 65) display the same pathological features while differing in some clinical features, as exemplified by onset symptoms (non-amnestic vs amnestic), progression rate (fast vs slow), involvement of hippocampus (relative preservation vs clear affection), CSF biomarkers (no clear difference), PET PiB (no clear difference) and PET FDG (no clear difference). The lack of these data in our study precludes further discussion of these issues.

In previous research, the reported number of subgroups has varied from two [51–55] to several [56, 57], according to the severity of disease, variations in the cognitive domains assessed, concomitant pathology (other degenerative and cerebrovascular diseases, inflammatory processes), brain resilience factors, size of the study sample (varying from population-based to hospital-based) and methods of defining the subgroups. The existence of subgroups in sAD has been defined by in vitro brain findings of neurofibrillary tangles [58], brain imaging of brain atrophy [59], or PET studies of tau levels [28, 54]. In these studies, the differentiation of subgroups was typically defined by the balance between cortical and medial temporal lobe brain involvement. If the medial temporal lobe was predominantly affected, it has been termed the limbic subtype, while if the cortex was predominantly affected, it has been termed the hippocampal-sparing subtype, often associated with early onset. The most typical form of AD, and the most common subtype of AD, encompassing about 75–80% of all AD cases, has been characterized by involvement of both cortical and medial temporal regions. To date, subgroups in AD have been characterized by empirical findings rather than by basic biological concepts in disease progression [54].

### Study characteristics

One limitation of this study was that the quadratic equations used to calculate YECO led to imaginary roots and missing data in some tests. However, most patients (63%) had valid data for all five tests and the majority of patients (98%) had valid data for at least three tests. Another limitation was that the sample may not be representative of typical memory clinic patients as the participants in this study were recruited to participate in PET research. Further, the assumption that all participants vary along an AD continuum may be incorrect, as some participants were PiB-. However, all except four participants were verified as having AD according to CSF criteria. One strength of our study is that a time scale for AD development was designed and validated in relation to biomarkers of AD. It would be possible to design a similar scale in other settings if cognitive data were known for adAD individuals, using equations relating cognition (or other parameters) to YECO and then applying these to sAD patients.

## Conclusions

A novel time scale of disease progression was designed based on cognitive functioning and validated both quantitatively and qualitatively. This measure of disease advancement was associated with biomarkers of AD; in contrast, there was no association between chronological age and AD biomarkers. The time scale made it possible to identify two AD subgroups, separated by time of disease onset; one was associated with an early onset, before 65 years of age, and the second was associated with a late onset, after 65 years of age. The early- and late-onset subgroups were differentiated by APOE e4 results, but not by CSF and PET biomarker results or cognitive profile.

## Supplementary Information

**Additional file 1: Figure 1.** Scatter plot of the relationship between chronological age at onset of disease and age at estimated onset of disease in patients with clinical diagnosis of MCI or AD.

## Data Availability

Anonymized data will be shared by request from any qualified investigator for the sole purpose of replicating procedures and results presented in the report given that data transfer agrees with EU legislation on the general data protection regulation.

## Abbreviations

PiB: ^11^C-Pittsburgh compound B
FDG: ^18^F-fluorodeoxyglucose
Aβ_42_: 42 Amino-variant of beta-amyloid
AD: Alzheimer’s disease
ATN: Amyloid/tau/neurodegeneration
adAD: Autosomal-dominant AD
DSM-V: Diagnostic and Statistical Manual of Mental Disorders
ICD-10: International Classification of Diseases
MCI: Mild cognitive impairment
NINCDS-ADRDA: National Institute of Neurological and Communicative Disorders and Stroke, and Alzheimer’s Disease and Related Disorders Association
p-tau: Phosphorylated tau181
RAVL: Rey Auditory Verbal Learning
RO retention: Rey-Osterrieth 30 min retention test
sAD: Sporadic AD
TMTA and TMTB: Trail Making tests
YECO: Years to expected (estimated) clinical onset
