# Supplementary figures and images for: A biomarker-validated time scale in years of disease progression has identified early- and late-onset subgroups in sporadic Alzheimer’s disease
Source: Alzheimers Res Ther. 2023 May 2;15:89. doi: 10.1186/s13195-023-01231-8 (PMC10152764; doi:10.1186/s13195-023-01231-8)

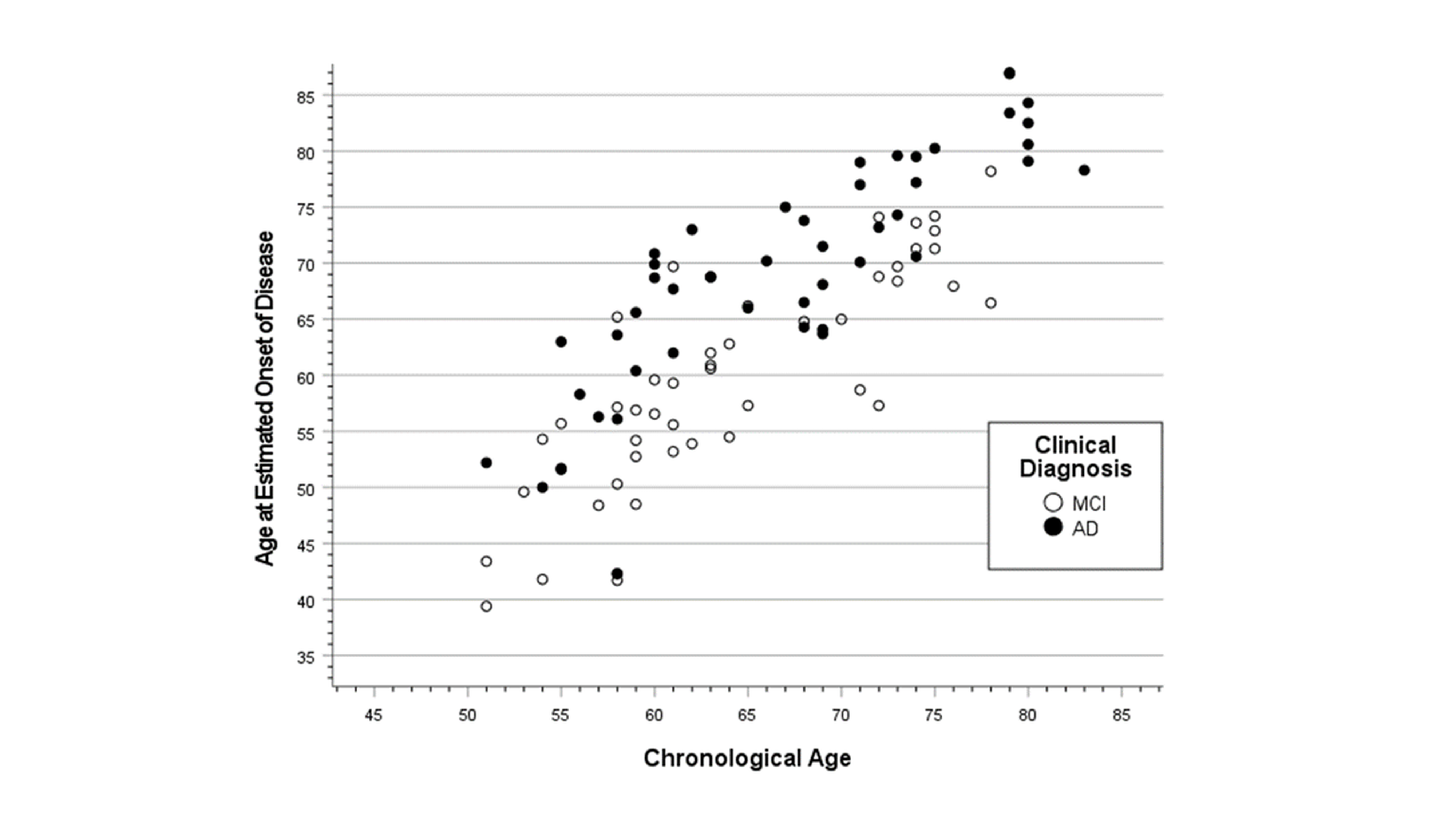

Supplement: Supplementary file 1 — Additional file 1: Figure 1. Scatter plot of the relationship between chronological age at onset of disease and age at estimated onset of disease in patients with clinical diagnosis of MCI or AD. [file 13195_2023_1231_MOESM1_ESM.tif]
